# Surgical Atrial Septal Patch Endocarditis in a Patient with a Complete Corrected Atrioventricular Canal Defect: A Case Report and Review of the Literature

**DOI:** 10.3390/diagnostics13050856

**Published:** 2023-02-23

**Authors:** Adela Serban, Alexandru Achim, Dana Elena Gavan, Raluca Tomoaia, Adrian Molnar, Mihai Suceveanu, Dan Damian Axente, Stefan Mot, Alexandra Dadarlat-Pop

**Affiliations:** 1 Cardiology Department, Heart Institute Niculae Stăncioiu, 19-21 Motilor Street, 400001 Cluj-Napoca, Romania; 2 5th Department of Internal Medicine, Faculty of Medicine, Iuliu Haţieganu University of Medicine and Pharmacy, 8 Victor Babes Street, 400012 Cluj-Napoca, Romania; 3 Clinical Rehabilitation Hospital, 46-50 Viilor Street, 400347 Cluj-Napoca, Romania; 4 Cardiovascular Surgery Department, Heart Institute Niculae Stăncioiu, 19-21 Motilor Street, 400001 Cluj-Napoca, Romania; 5 7th Department of Surgery, Faculty of Medicine, “Iuliu Hatieganu” University of Medicine and Pharmacy, 400012 Cluj-Napoca, Romania; 6 Cluj-Napoca Municipal Clinical Hospital, 400139 Cluj-Napoca, Romania

**Keywords:** congenital heart disease, atrioventricular canal defect, surgical atrial patch, infectious endocarditis

## Abstract

Infective endocarditis (IE) is common in patients with corrected congenital heart disease (CHD) with a residual lesion, but is rarely found on surgical patches used to close atrial septal defects (ASDs). This is also reflected in the current guidelines that do not recommend antibiotic therapy for patients with a repaired ASD with no residual shunt six months after closure (percutaneous or surgical). However, the situation could be different in the case of mitral valve endocarditis, which causes leaflet disruption with severe mitral insufficiency and could seed the surgical patch. We present herein a 40-year-old male patient with a past medical history of a complete surgically corrected atrioventricular canal defect performed in childhood who presented with fever, dyspnea and severe abdominal pain. Transthoracic and transesophageal echocardiography (TTE and TEE) revealed vegetation at the level of the mitral valve and the interatrial septum. The CT scan confirmed ASD patch endocarditis and multiple septic emboli, guiding the therapeutic management. An accurate evaluation of cardiac structures should be mandatory when a systemic infection is detected in CHD patients, even if the defects were surgically corrected, because the detection and eradication of such infectious foci as well as a surgical reintervention are particularly difficult to achieve in this subpopulation.

## 1. Introduction

The overall incidence of infective endocarditis (IE) in patients with congenital heart disease (CHD) is significantly higher than in the general population [1]. IE incidence is strongly correlated with age also among patients with CHD, as the majority of IE cases are seen in older patients with CHD [2,3]. However, IE mainly occurs in unrepaired cyanotic congenital heart defects and in repaired defects with residual shunt or valvular regurgitation. Even if not repaired, due to the slow velocity of shunt flow, an atrial septal defect (ASD) has a very low risk of IE and therefore antimicrobial prophylaxis is recommended (according to the guidelines) only in patients with an ASD who have a prior history of IE or a residual shunt adjacent to a prosthetic patch or prosthetic device or during the six months after closure [4]. Infections of the surgical atrial septal patch are rarely seen and late IE (after 6 months) at this level is even rarer. Early IE on prosthetic patches or prosthetic devices is associated with incomplete endothelization [4], while in late IE cases, underlying mitral valvulopathy with subsequent severe mitral valve destruction has been noted, perhaps explaining a two-step mechanism of bacterial colonization from the mitral valve to the nearby interatrial septum [5].

Atrial septal defects larger than 1 cm require percutaneous or surgical closure. Previously, surgical closure was the standard of care for ASD, but over the last 30 years, transcatheter devices have rapidly emerged as the gold standard for ostium secundum ASD [6]. Infectious complications of atrial septal occluder devices represents about 0.1% of cases [7]. The risk of infectious endocarditis on the surgical patch is usually related to the time of surgery; the risk is higher in the first 3 months. Infection of a percutaneous closing device can occur either in association with the procedure, involving microorganism inoculation, or by later hematogenous spread [8,9].

We present a case of a 40-year-old male patient with a past medical history of a complete atrioventricular canal defect surgically corrected in his childhood who presented with late IE of the mitral valve and the surgical patch. Multimodal imaging led to the diagnosis and the discovery of multi-organ septic embolizations. We reviewed the literature to find similar reports in order to understand the evolution and the therapeutic management of these patients.

## 2. Case Report

A 40-year-old male was admitted to the emergency department for chills, a fever of 39 °C, dyspnea, severe abdominal pain and fatigue lasting 9 days. The fever started insidiously, the abdominal pain appeared suddenly 3 days prior to admission and the dyspnea got progressively worse, forcing the patient to seek medical help. He denied any history of travel or exposure to animals. His COVID-19 test was negative on admission. The family and social histories were noncontributory.

In the patient’s medical history, he had undergone surgery for a complete atrioventricular canal defect at 7 years old. The ostium primum septal defect was closed by a Dacron patch and the anterior mitral valve cleft was corrected by a cleft suture. After surgery, moderate residual mitral regurgitation was detected on several echocardiographic assessments, with no prior evidence of a leak over the atrial septum.

On physical examination, he was tachycardic and tachypneic, with a systolic murmur, bilateral fine rales, peripheral oedema, jaundice and also Osler nodules on his hands.

A laboratory analysis demonstrated severe inflammatory syndrome (WBC = 21 × 103/µL, CRP = 213 mg/L and Feritine = 3444 µg/L), elevated NTproBNP (=1874 pg/mL) and hepatic dysfunction (GPT = 215 UI/L and GOT = 142 UI/L). The blood cultures were positive for methicillin-sensitive Staphylococcus aureus (MSSA). Two cultures at an interval of 12 h were found positive with the same germ. He presented no risk factors for community *Staphylococcus aureus* infection (skin abrasions, wounds, etc.) other than acne.

The ECG showed sinus tachycardia with left axis deviation and a RSR pattern in V1, which is characteristic of the ostium primum defect.

**Figure 1:**
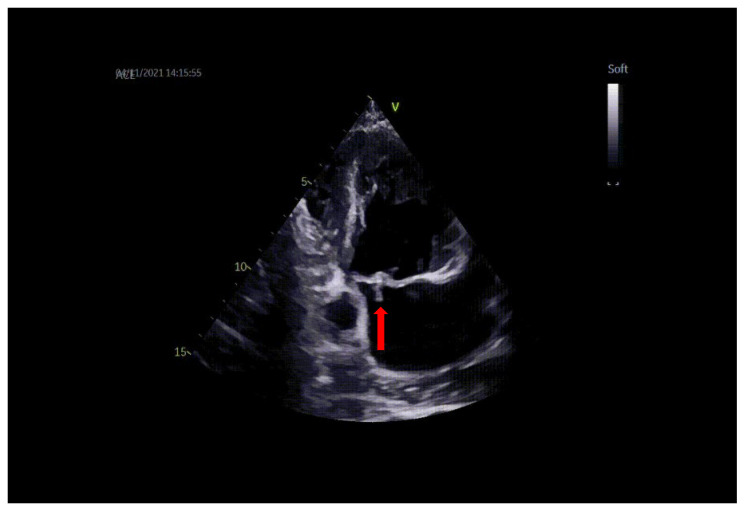
TTE: vegetation on the mitral valve prolapsing in LA (arrow).

**Figure 2:**
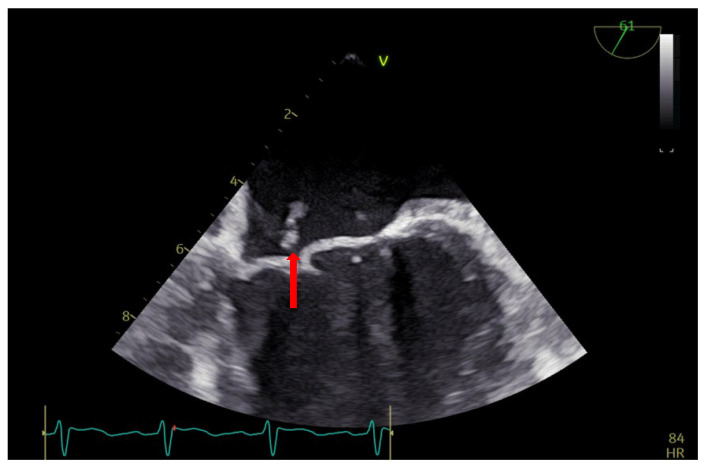
TEE: vegetation on the anterior mitral valve in the cleft area (arrow).

**Figure 3:**
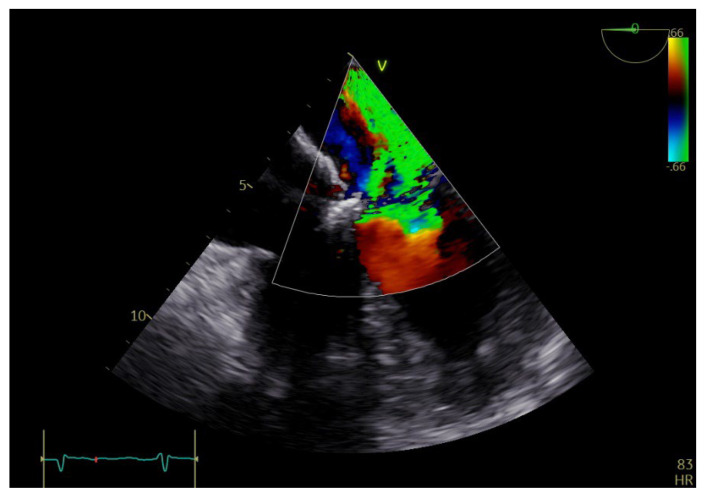
TEE: color Doppler revealing severe mitral regurgitation.

Transthoracic and transesophageal echocardiography (TTE and TEE) revealed a thickened anterior mitral valve in the cleft area, with a highly mobile echogenic structure about 10 mm prolapsing in the left atrium (LA) (Figure 1 and Figure 2), suggestive of vegetation with high embolic risk. Severe mitral regurgitation was detected on color Doppler evaluation with the jet origin in A2–A3 mitral segments (Figure 3).

**Figure 4:**
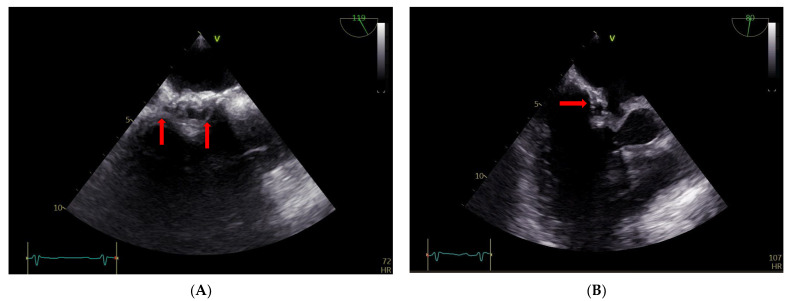
(**A**,**B**) TEE: abscess on the right side of the atrial surgical patch with ecolucent spaces (arrows).

The TEE findings revealed that the atrial septum was infiltrated, particularly on the right side, and thickened, with mobile structures of about 5 mm in diameter (Figure 4, Panel A). There were also some round ecolucent spaces with a color Doppler signal, indicative of an abscess (Figure 4, panel B).

**Figure 5:**
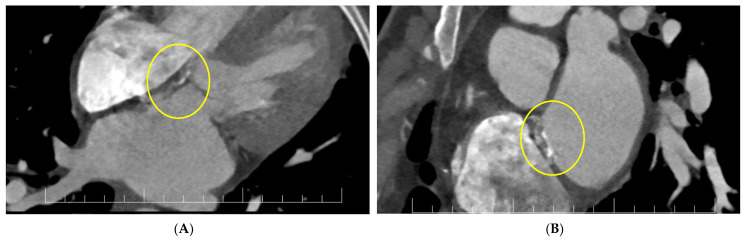
(**A**,**B**) Cardiac CT: interatrial septal patch abscess with a fistulous path (inside circles).

For a better description of the atrial septum, a cardiac CT was performed. This confirmed the presence of the vegetation at the level of the anterior mitral valve and revealed diffused hypocaptant thickening at the interatrial septum with a fistulous path (Figure 5).

Thus, the diagnosis of infective endocarditis was confirmed by two major criteria: blood cultures and an echocardiography positive for mitral valve vegetation and patch fistula.

**Figure 6:**
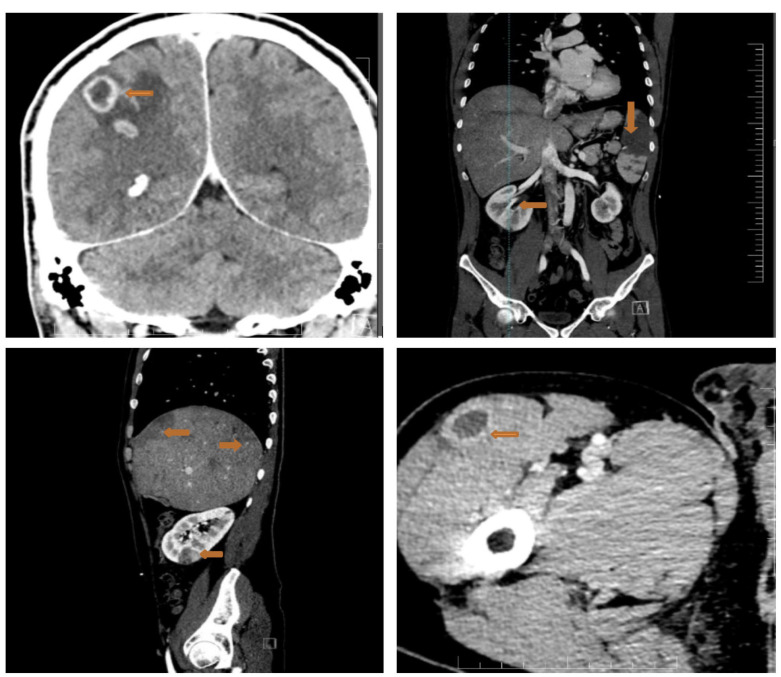
Full-body CT scan showing multiple septic emboli. Upper left panel: the arrow shows abscesses at the cerebral level (brain abscesses: ring enhancing lesions with slightly perilesional oedema); upper right panel: large splenic abscess (arrow); lower left panel: liver structure with multiple subcapsular peripheral areas, imprecisely delimited with low contrast uptake (budding abscesses) (arrows), plus a right renal abscess (arrow); right lower panel: pelvic muscle abscess (arrow).

Considering the high embolic risk of the mitral vegetation, we proceeded with a full-body CT scan that showed multiple septic emboli interpreted as abscesses at the cerebral, splenic, hepatic, renal and pelvic muscle level (Figure 6).

The size of the mitral valve vegetation could represent only a remnant of the initial vegetation because of several embolizations.

The endocarditis team emphasized the need for cardiac surgery, i.e., repair or replacement of the mitral valve and a patch replacement for the atrial septum, and an urgent need to start antibiotic treatment with oxacillin, rifampicin and gentamicin.

Brain abscesses were clinically silent and did not require surgical treatment, only neurological and CT monitoring.

After two weeks of antibiotic treatment, the clinical course was unfavorable, with a persistent infection, fever and inflammatory syndrome; therefore, we repeated the CT scan, which showed the extension of the splenic abscess. Consequently, we proceeded to a splenectomy, which was without complications.

The hepatic abscesses, which explained the initial symptomatology with severe abdominal pain, developed favorably and decreased in number and extent.

After 56 days of hospitalization and antibiotic treatment, the CT scan revealed resorption of the cerebral, renal, hepatic and muscle abscesses.

The patient was discharged in good clinical and biological condition. The triple antibiotic therapy lasted for 6 weeks and the blood cultures became negative after 1 week. Although the mitral valve had severe regurgitation and the atrial patch had an abscess and a fistula, the patient refused cardiac reintervention. Although the entire multidisciplinary team advocated surgical treatment, the patient categorically refused it. Abscess sterilization remains an open issue, the outcome of which is difficult to predict or control. The patient decided to check their evolution with CT and to initiate any additional antibiotic therapy depending on their clinical condition. At the 4-month follow-up, he had no further hospitalizations or cardio-embolic events. The latest imagistic follow-up was at this time, showing no organ abscesses but with persistent vegetation at the level of the surgical patch.

## 3. Discussion

### 3.1. Current Reports and Actual Evidence

It is well known that the risk of infective endocarditis is 15–140 times higher for patients with congenital heart defects than in the rest of the population [3,10]. Moreover, it has recently been shown that there is clear delay in establishing IE diagnosis amongst CHD patients in Central and South-Eastern European countries [11]. However, relative to other congenital diseases, the association between ASD and IE is very rare, at only around 0.4% [12,13].

From a surgical point of view, there are two main sources of traditional patches for repairing intracardiac defects. One is the auto pericardial patch, with a good histocompatibility and tissue activity, but it is inaccessible and weak. The other one is the Dacron patch, which has a thin texture and a strong tension and elasticity, and is prone to anastomotic deformation, thrombosis, embolism, hemolysis and infection after repair [14].

In the CONCOR registry, the incidence rate of IE in ASD was increased by the presence of other lesions that rendered them vulnerable to IE, for example, either valvular, particularly on the mitral valve, or small ventricular septal defects [15].

Removal of all infected tissue is the major objective of early surgery in IE. The published European recommendations on surgical indications for IE do not strictly apply to the population of children and adults with CHD [16]. To date, there has been no explicit recommendation for these patients. The timing of IE surgery has shifted to early intervention. In a randomized study, Kang et al. found that surgery as soon as 2 days after diagnosis resulted in a lower rate of death, embolic events or recurrence of IE after 6 months [17]. Surgery during the first 7 days has been linked to a lower rate of mortality [18].

As in our case, the residual mitral regurgitation after cleft repair was in the initial lesion where the vegetation was attached, and from here the infection spread to the interatrial septum patch. The fact that the patient remained with residual mitral insufficiency after the operation argues in favor of the cleft area being only partially sutured and it being the most likely initial area of bacterial seeding. Indeed, a population-based study following up patients with CHD for IE for up to 30 years reported that the incidence of endocarditis after repair of primum ASD with a mitral cleft was 1.8/1000 patient-years, carrying a moderate to low risk, while the incidence of endocarditis after repair of secundum ASD was zero [19]. However, in a study by Morris et al., a significant proportion of patients were lost at follow-up [19]. Contrastingly, Snygg-Martin et al. reported an 8.5% cumulative incidence of CHD IE at 87 years of age compared with 0.7% in matched controls, but with the lowest IE incidence was in patients with ASD (27.8 per 100,000 person-years) [3]. These findings reflect the rarity of these cases. The particularity of our case consists of the consecutive seeding of the atrial patch many years apart, when the patch was completely endothelialized and, regardless, all of the interatrial septum was a poorly vascularized structure.

In a study focusing on 13 autopsy cases of Dacron patch IE, the occurrence of IE on the surgical patch of the interatrial septal defect was detected in only one patient; the rest suffered from IE of the patch covering the interventricular septum [20]. The IE was more often found early than late, at approximately 30 days postoperatively, and was associated with a sternal site infection in 8 of the 10 patients [20]. Their latest case of IE occurred about 4 months after operation [20], and practically all cases were considered as periprocedural IE—a fact that gives our case a peculiarity. Interestingly, in 77% of cases, IE was not isolated on the Dacron patch but involved a neighboring cardiac structure (aortic valve, mitral valve, tricuspid valve, etc.) [20]. In 55% of cases, the incriminating bacteria was *Staphylococcus aureus* [20], in accordance with a parallel review of 21 cases of IE of percutaneous ASD closure devices, where 57% of patients had the same bacteria in their blood cultures [21].

In any case, correction of a congenital defect (especially with residual lesions), either simple or complex, carries an inevitable risk of bacterial colonization of the new structures, which is difficult to treat and diagnose and occasionally leads to IE [20]. In some cases (such as our report), medical treatment is unlikely to be able to eradicate infection without surgery.

We found a similar case of tardive IE on the surgical patch on the ASD in the literature. This was associated with a cerebral abscess which, unlike our case, required surgical drainage. TEE showed a shunt at atrial level suggestive of atrial septal patch dehiscence without vegetation. In this case, the diagnosis was completed with 18 F- FDG-PET/CT, which detected the existence of a metabolic hyperactivity compatible with interatrial septal endocarditis [5].

Regarding the brain abscesses, surprisingly, the neurological evolution in this case was completely asymptomatic, with the brain abscess resorbed under antibiotic treatment. Brain examinations, either CT or IRM, are crucial for the diagnosis of brain abscesses, as they are often clinically silent but can significantly worsen the prognosis [22,23,24]. In case of brain abscesses, surgery is often delayed and based on serial imaging and clinical progression, as the treatment for brain abscess is first and foremost with antibiotics to include MRSA coverage if the organism is not known [25].

The etiology with MSSA is positively correlated with the formation of emboli, most frequently cerebral, and mortality due to specific virulence factors and also its ability to elicit extensive myocardial tissue destruction [26]. The case of our patient has shown that antibiotic therapy can eliminate multiple septic metastases, and that in patients in whom surgery is associated with high-risk or is refused, a “conservative” treatment actually addresses the etiopathogenetic cause of this particular germ. This of course comes with a significant and extremely unpredictable trade-off; namely, the risk of relapse and irreversible destruction of some cardiac structures.

Finally, regarding the potential portals of entry of MSSA, skin infection represents the most possible cause. It is well known that in approximately one-half of patients with MSSA, no portal of entry can be documented. In this case, acne represented the only possible entry that could be detected [27]. “Hidden” entry portals for IE could be discrete skin lesions in patients with diabetes mellitus and peripheral artery disease [28] or micro-organisms of gastrointestinal origin (gastrointestinal polyps, tumors, etc.) [29].

We presented this rare case of surgical atrial patch endocarditis, which we diagnosed based on the TEE exam, actually starting from the TTE that showed vegetation at the level of the mitral valve. As a result of the multiple septic emboli, the size of the vegetation at the time of presentation could represent only a remnant of the original vegetation. During the TEE examination, there was an insignificant hemodynamic shunt at the atrial level, which appeared to be as a result of endocarditis, in contrast to previous TEEs, where the appearance of the septum/patch was linear, thin, and uniform, without a residual shunt. In the current TEE, the septum was thickened with echo-lucent spaces and mobile formations which, in the clinical context of IE, we interpreted as vegetations, abscesses and fistulous tracts.

Although the CT examination does not provide additional information regarding the valvular damage in endocarditis, it was essential in this case for the diagnosis of atrial septal patch infection in addition to the description of the multiple septic emboli. Indeed, after the 6-week-course of antibiotics, a cardiac MRI could have evaluated more precisely the size of the fistula and the level of inflammation around the affected structures, or a PET/CT at follow-up could have evaluated any hyperactivity.

In this case, with infection at the atrioventricular level and extension into the ostium primum area of the atrial septum, a surgical approach was considered extremely difficult due to the need to replace the mitral valve and the patch in conditions of swollen, friable and infected tissue and the particular anatomy of the atrioventricular canal defect.

We focused mainly on the imaging diagnosis, precisely because of the rarity of this type of endocarditis and also because of the role of multimodality imaging in the complete evaluation and deciding the therapeutic management of this complex case.

### 3.2. Future Outlooks

The authors acknowledge that mitral valve and early corrected ASD IE are not new pathologies, and indeed, similar cases have been reported. The interplay between the two structures has been demonstrated, but very late IE of a healed septal patch (decades apart) involves a different bacterial seeding mechanism, and the cases are extremely rare with surgical corrections [5] and slightly more common with occluded devices [30,31,32], probably due to partial endothelization of the metal frame. The novelty of our case comes from the interval of more than 30 years between the operation and the infective episode and that, until the present time, it was known that surgical patch closure of atrial defects does not pose a risk for IE. Undoubtedly, residual valvulopathies play an important role [5,30], but the seeding risk and mechanism of the atrial patch remains unknown. In patients with partial or complete atrioventricular canal defects and mitral clefts, residual mitral insufficiency can occur immediately after surgery or at a distance due to the degeneration of the sutures at the level of the malformed valve, which automatically increases the risk of IE. In the present case, during the follow-up, a re-intervention was recommended to the patient to correct or replace the mitral valve.

There are no guidelines for the prevention, diagnosis or management of this complication. Re-intervention is particularly demanding and complete medical sterilization is almost impossible to achieve. Injecting a radiolabeled analog of glucose during PET/CT may be indicated in all corrected CHD patients with suspected IE to detect any inflammatory leukocytes on the corrected cardiac structures early. Considering the late infectious episode (33 years after the intervention), we recommend the implementation of IE prophylaxis even in the absence of residual atrial shunt and residual mitral insufficiency in this category of patients. The deficit in the embryonic development of the heart skeleton (endocardial cushion defect) can favor the propagation of the mitral valve–atrial septum infection, making curative surgical re-intervention of the extensive infection extremely difficult.

## 4. Conclusions

IE should always be suspected in patients with fever and corrected CHD. In our patient, the complete corrected atrioventricular canal defect with residual mitral regurgitation represented the initial infected tissue lesion.

Multimodality imaging (TTE, TEE and cardiac CT) is essential in the diagnosis and management of surgical atrial patch endocarditis.

## Data Availability

Not applicable.
